# Patients with Primary Immunodeficiencies Are a Reservoir of Poliovirus and a Risk to Polio Eradication

**DOI:** 10.3389/fimmu.2017.00685

**Published:** 2017-06-13

**Authors:** Asghar Aghamohammadi, Hassan Abolhassani, Necil Kutukculer, Steve G. Wassilak, Mark A. Pallansch, Samantha Kluglein, Jessica Quinn, Roland W. Sutter, Xiaochuan Wang, Ozden Sanal, Tatiana Latysheva, Aydan Ikinciogullari, Ewa Bernatowska, Irina A. Tuzankina, Beatriz T. Costa-Carvalho, Jose Luis Franco, Raz Somech, Elif Karakoc-Aydiner, Surjit Singh, Liliana Bezrodnik, Francisco J. Espinosa-Rosales, Anna Shcherbina, Yu-Lung Lau, Shigeaki Nonoyama, Fred Modell, Vicki Modell, The JMF Centers Network Investigators and Study Collaborators, Ahmet Ozen, Mohamed-Ridha Barbouche, Mark A. McKinlay

**Affiliations:** ^1^Research Center for Immunodeficiencies, Pediatrics Center of Excellence, Children’s Medical Center, Tehran University of Medical Science, Tehran, Iran; ^2^Faculty of Medicine, Department of Pediatric Immunology, Ege University, Izmir, Turkey; ^3^Global Immunization Division, Centers for Disease Control and Prevention, Atlanta, GA, United States; ^4^Division of Viral Diseases, Centers for Disease Control and Prevention, Atlanta, GA, United States; ^5^Center for Vaccine Equity, Task Force for Global Health, Atlanta, GA, United States; ^6^Jeffrey Modell Foundation, New York, NY, United States; ^7^Research and Product Development, World Health Organization, Geneva, Switzerland; ^8^Department of Clinical Immunology, Children’s Hospital of Fudan University, Shanghai, China; ^9^Division of Immunology, Department of Pediatrics, Hacettepe University Faculty of Medicine, Ankara, Turkey; ^10^Department of Allergology and Immunotherapy, Institute of Immunology, Moscow, Russia; ^11^Department of Pediatric Immunology and Allergy, Ankara University School of Medicine, Ankara, Turkey; ^12^Department of Clinical Immunology, The Children’s Memorial Health Institute, Warsaw, Poland; ^13^Institute of Immunology and Physiology, Ural Branch of the Russian Academy of Sciences, Yekaterinburg, Russia; ^14^Department of Pediatrics, Federal University of São Paulo, São Paulo, Brazil; ^15^Grupo de Inmunodeficiencias Primarias, Facultad de Medicina, Departamento de Microbiología y Parasitología, Universidad de Antioquia, Medellín, Colombia; ^16^Pediatric Department A and the Immunology Service, Sheba Medical Center, Tel Hashomer, Jeffrey Modell Foundation Center, Affiliated to the Sackler Faculty of Medicine, Tel Aviv University, Tel Aviv, Israel; ^17^Division of Pediatric Allergy and Immunology, Marmara Medical Faculty, Istanbul, Turkey; ^18^Pediatric Allergy and Immunology Unit, Advanced Pediatrics Centre, PGIMER, Chandigarh, India; ^19^Dr. Ricardo Gutierrez Hospital de Niños, Buenos Aires, Argentina; ^20^Clinical Immunology and Allergy Unit, Instituto Nacional de Pediatría, Ciudad de México, Mexico; ^21^Department of Clinical Immunology, Dmitry Rogachev Federal Research and Clinical Center of Pediatric Hematology, Oncology and Immunology, Moscow, Russia; ^22^Department of Paediatrics and Adolescent Medicine, The University of Hong Kong, Queen Mary Hospital, Hong Kong, Hong Kong; ^23^Shenzhen Primary Immunodeficiency Diagnostic and Therapeutic Laboratory, Hong Kong University-Shenzhen Hospital, Shenzhen, China; ^24^Department of Pediatrics, National Defense Medical College, Saitama, Japan; ^25^Department of Immunology, Institut Pasteur de Tunis, University Tunis El-Manar, Tunis, Tunisia

**Keywords:** poliovirus eradication, immunodeficiency-associated vaccine-derived polioviruses, oral poliovirus vaccine, humoral immunodeficiency, combined immunodeficiency, primary immunodeficiency

## Abstract

Immunodeficiency-associated vaccine-derived polioviruses (iVDPVs) have been isolated from primary immunodeficiency (PID) patients exposed to oral poliovirus vaccine (OPV). Patients may excrete poliovirus strains for months or years; the excreted viruses are frequently highly divergent from the parental OPV and have been shown to be as neurovirulent as wild virus. Thus, these patients represent a potential reservoir for transmission of neurovirulent polioviruses in the post-eradication era. In support of WHO recommendations to better estimate the prevalence of poliovirus excreters among PIDs and characterize genetic evolution of these strains, 635 patients including 570 with primary antibody deficiencies and 65 combined immunodeficiencies were studied from 13 OPV-using countries. Two stool samples were collected over 4 days, tested for enterovirus, and the poliovirus positive samples were sequenced. Thirteen patients (2%) excreted polioviruses, most for less than 2 months following identification of infection. Five (0.8%) were classified as iVDPVs (only in combined immunodeficiencies and mostly poliovirus serotype 2). Non-polio enteroviruses were detected in 30 patients (4.7%). Patients with combined immunodeficiencies had increased risk of delayed poliovirus clearance compared to primary antibody deficiencies. Usually, iVDPV was detected in subjects with combined immunodeficiencies in a short period of time after OPV exposure, most for less than 6 months. Surveillance for poliovirus excretion among PID patients should be reinforced until polio eradication is certified and the use of OPV is stopped. Survival rates among PID patients are improving in lower and middle income countries, and iVDPV excreters are identified more frequently. Antivirals or enhanced immunotherapies presently in development represent the only potential means to manage the treatment of prolonged excreters and the risk they present to the polio endgame.

## Introduction

Primary immunodeficiencies (PIDs) are a heterogeneous group of inherited disorders due to developmental defects or dysfunction of the immune system components (1). PID patients can potentially be infected by immunizations if they receive live vaccines (2). Attenuated oral poliovirus vaccine (OPV) immunization has been associated with poliovirus infection in patients with primary antibody deficiencies and combined immunodeficiencies, which can lead to paralysis (3–5).

Paralysis is not the only risk of OPV immunizations in PID patients since some vaccinated PID patients may shed vaccine-derived polioviruses (VDPVs) due to a prolonged period of intestinal replication. VDPV variants of OPV serotypes (PV1, PV2, and PV3) show increased nucleotide divergence in the viral protein 1 (VP1) coding region associated with increased neuropathogenicity (6). Although neonatal screening has begun to expedite the early diagnosis of patients with severe combined immunodeficiencies (SCIDs) and agammaglobulinemia to modify their vaccination program and therapeutic management, these screening tests do not diagnose T or B cell dysfunction, nor have they been implemented worldwide. On the other hand, approximately 150 countries still use OPV in the national childhood immunization schedule, which can spread and be transmitted to PID patients incidentally. Approximately 100 VDPV infections have been reported in PID patients worldwide to date (4, 7, 8). As a potential reservoir for neurovirulent VDPV strains, infected PID patients represent a global risk to unimmunized contacts and to the Global Polio Eradication Initiative (4, 9).

The significant impact of OPV on the elimination of poliomyelitis due to wild poliovirus (WPV) and its additional beneficial properties (e.g., economic costs, easy administration, and superior mucosal antibody response) are evident. However, because WPV type 2 has been eradicated and 90% of circulating VDPV, and approximately 40% of VAPP cases are caused by type 2, type 2 was removed from OPV in all countries globally in April 2016. As a result, screening for immunodeficiency-associated VDPV (iVDPV) shedding of known PID patients is more critical to completing elimination of live type 2 poliovirus from the world (10, 11).

Thus, assessing the risk associated with prolonged iVDPV excretion among PID patients by estimating the prevalence in a worldwide study is of critical importance for stakeholders and decision-makers to build an effective strategy for the polio endgame, including development of potential treatments such as antivirals. Based on several reports showing that not only patients with primary antibody deficiencies but also combined immunodeficiencies are very susceptible to persistent polio and non-polio enterovirus (NPEV) infections (4, 12), this multicenter study has been designed to determine the prevalence of iVDPV in patients with both types of immunodeficiencies and characterize the genetic properties of associated virus strains.

## Materials and Methods

### Patients

All patients enrolled in this study were diagnosed with PID from 19 Jeffrey Modell Foundation (JMF) sites in 13 OPV-using countries from January 2014 to November 2015 (Table 1). Inclusion criteria encompassed patients 6 weeks of age or greater and having a diagnosis of SCID, combined immunodeficiency, agammaglobulinemia, or common variable immunodeficiency (CVID). Other types of PID patients were not included in this study.

**Table 1:** 19 Jeffrey Modell Foundation sites from 13 countries enrolled PID patients.

Country^a^	City	Site no.^b^	Enrolled patients	Culture results	National polio vaccination^c^	PID screening
India	Chandigarh	Site 30	23	23	5 OPV doses (birth-6w-10w-14w-16m)	No

Tunisia	Tunis	Site 12	40	40	1 IPV dose (6m)	No
					7 OPV doses (2m-3m-6m-18m-6y-12y-18y)	

China	Shanghai	Site 23	52	51	4 OPV doses (2m-3m-4m-4y)	No
	Hong Kong	Site 21	11	11	6 OPV doses before 2007 (birth-3m-5m-18m-6y-11y)	No

Colombia	Medellin	Site 24	25	25	5 OPV doses (2m-4m-6m-18m-5y)	No

Iran	Tehran	Site 14	102	102	6 OPV doses (birth-2m-4m-6m-18m-6y)	No

Mexico	Mexico City	Site 25	20	20	2 OPV doses (>6m-<5y)	No

Poland	Warsaw	Site 17	29	29	3 IPV doses (3m-5m-16m)	No
					1 OPV dose (6y)	

Russia	Moscow	Site 18	35	35	2 IPV doses (3m-4.5m)	No
					4 OPV doses (6m-18m-20m-14y)	
	Moscow	Site 28	20	20		
	Yekaterinburg	Site 19	28	28		

Turkey	Ankara	Site 27	30	29	2 IPV doses (2m-4m)	No
					2 OPV doses (6m-18m)	
	Ankara	Site 26	43	43		
	Izmir	Site 11	75	75		
	Istanbul	Site 20	24	24		

Israel	Tel Hashomer	Site 29	24	24	2 OPV doses (6m-18m)	No

Japan	Tokyo	Site 13	9	8	2 OPV doses before 2012 (3m-18m)	No

*OPV, oral poliovirus vaccine; IPV, inactivated polio vaccine; PID, primary immunodeficiency; w, weeks; m, months; y, years*.

*^a^Countries are organized in order of World Bank income classification (lower middle, upper middle, and upper income)*.

*^b^Site numbers are provided to correspond with subject numbers in Tables 3 and 4*.

*^c^WHO vaccine-preventable diseases: monitoring system. 2016 Global summary*.

Assessment of each patient at the different JMF sites met the updated criteria introduced by the European Society for Immunodeficiencies^1^ (13) and/or the American Academy of Allergy, Asthma & Immunology for the diagnosis of PID (14). In all studied cases, other defined secondary causes of immunodeficiency were excluded. Other types of PID patients were not included in this study. The standard criteria for diagnosis of patients were summarized in Table S1 in Supplementary Material.

Written informed consent (if 18 years of age or older), assent (if 7–17 years of age) or consent from the parents/guardian (for those less than 7 years) was obtained, in accordance with the principles of the ethics committees or Institutional Review Board of the local institutes using common forms designed by The Task Force for Global Health (TFGH, protocol no: CVE-001, WIRB^®^ Protocol #20130957; Data Sheet S1 in Supplementary Material) translated to the participant JMF sites’ local languages.

### Study Design

Each selected JMF study site identified patients who met the inclusion criteria at the time of enrollment. After collecting consent or assent forms, the JMF study site collected the relevant information to complete the patient’s log (to track enrollment and to maintain the link between the unique identifiers) and case report form (CRF), and assigned a coded identification number (ID) under a standardized procedure. IDs, names, and contact information was recorded in the patient’s log, which was stored securely at the site until completion of the study. All specimens and CRFs were labeled with ID only and not any personal identifiers. The CRF included region (country based on JMF site), demographic characteristics (including date of birth and gender), clinical features (type of PID, age of onset and medical records including signs or symptoms of poliovirus infection), polio vaccination history (date of first and last OPV exposure and type of exposure), PID treatment modalities, and specimen collection information.

Each JMF study site chose the optimal procedure for collection of two stool samples (about 8 g using provided kits) over a 4-day period depending on the proximity of the patient to the JMF study site, the practicality of patient travel to the study site and the availability of adequate cold storage of the stool sample (Table S2 in Supplementary Material).

After collection, the stool samples were kept refrigerated at a temperature of 2–8°C and shipped to the local or regional Global Polio Laboratory Network (GPLN) laboratory along with supporting documentation. The JMF study site provided an electronic copy of the CRF by encrypted means (using a secure FTP server at TFGH) to Centers for Disease Control and Prevention (CDC) for secure data management. Stool specimens were sent to the GPLN laboratory for isolation of poliovirus and NPEVs. Isolates were sent to the CDC polio/enterovirus laboratory using standard shipping protocols for further characterization and genomic sequencing. GPLN member laboratories in all six WHO regions followed standardized protocols to identify and isolate poliovirus and NPEV. They also differentiated the three poliovirus serotypes, Sabin-like poliovirus, and vaccine derived (VDPV). For VDPVs, they conducted genomic sequencing to determine how long the virus had been circulating or had been excreted in the case of iVDPV excreters. This was done by comparing the nucleotide sequence of the VP1 region of the genome from PV isolates according to published protocols (15, 16). The number of mutations compared to the parent Sabin strain is approximately proportional to the duration of excretion. The accepted definition of an iVDPV is >1% nucleotide divergence from Sabin types 1 and 3 or >0.6% for Sabin type 2 in the VP1 coding region (6).

If testing results were positive for poliovirus or NPEV, the JMF site physician assured that family members were appropriately vaccinated against polio and educated about principles of good hygiene to prevent fecal–oral transmission. The physician also followed-up any patient found to be excreting poliovirus periodically (every 1–2 months) until the cessation of excretion and the procedures for testing and reporting above were followed each time. Patients were considered as having cleared an infection if two consecutive stool specimens in 2 months were negative according to the WHO-recommended surveillance standard of poliomyelitis.^2^

The TFGH study manager ensured coordination of specimen shipment along with the JMF scientific program director, and laboratory results were communicated to CDC both by the GPLN laboratory through the GPLN global coordinator and by the TFGH study manager and entered into the secure database at CDC.

### Statistical Analysis

Statistical analysis was performed using a commercially available software package (SPSS Statistics 17.0.0, SPSS, Chicago, IL, USA). The one-sample Kolmogorov–Smirnov test was applied to estimate whether data distribution is normal. Parametric and non-parametric analyses were performed based on the finding of this evaluation. A *p*-value of 0.05 or less was considered statistically significant.

## Results

### Characteristics of Studied PID Cases and OPV Vaccination

During the study period, a total of 635 patients (444 males and 191 females) were included into this study. CVID was the most common diagnosis of studied patients (*n* = 320) followed by agammaglobulinemia (*n* = 250), SCID (*n* = 54) and combined immunodeficiency [*n* = 11; all with a diagnosis of major histocompatibility complex (MHC) class II deficiency]. Age at time of study enrollment was 21.1 ± 15.7 years (mean ± SD) for patients with CVID, 18.0 ± 8.5 years for patients with agammaglobulinemia, 7.9 ± 7.8 years for patients with MHC class II deficiency, and 3.8 ± 4.9 years for SCID patients. None of the patients with SCID or MHC class II deficiency were transplanted prior to the study entry.

Among all enrolled patients from these OPV-using countries, 44 individuals did not receive OPV vaccination based on the prior family history of PID and early diagnosis or modified inactivated polio vaccine-based national childhood immunization schedule (Table 2). However, the median age at first OPV dose was approximately 2 months (range from birth to 6 years) for the 584 patients who did receive OPV. Past medical history of paralytic disease was identified in 14 patients (1.25% of CVID and 4% of agammaglobulinemia patients), but none of the SCID or MHC class II deficiency cases had a history of paralytic disease. Agammaglobulinemia patients were younger than SCID and CVID patients when they received their last dose of OPV, consistent with the cessation of further OPV doses after an earlier clinical diagnosis of PID (Table 2).

**Table 2:** Demographic data summary for all 635 PID patients.

Parameters	All patients	CVID	Agammaglobulinemia	SCID	MHC class II deficiency
Number of patients	635	320	250	54	11
Gender (M/F)	444/191	179/141	237/13	24/30	4/7
Current age, mean; years (SD)	12.7 (8.2)	21.1 (15.7)	18.0 (8.5)	3.8 (4.9)	7.9 (7.8)
Age at first OPV dose, mean; years (SD)	0.6 (0.8)	0.5 (1.6)	0.4 (1.2)	1.0 (2.6)	0.6 (0.9)
Age at last dose, mean; years (SD)	2.3 (2.0)	4.3 (4.9)	3.0 (3.6)	2.2 (3.4)	NA
Exposure to OPV (Y/N)	584/51	303/17	233/17	37/17	11/0
Paralytic disease	14	4	10	0	0

*OPV, oral poliovirus vaccine; CVID, common variable immune deficiency; SCID, severe combined immunodeficiency; PID, primary immunodeficiency; MHC, major histocompatibility complex*.

### Use of Intravenous and Subcutaneous Immunoglobulin

The majority (97%) of enrolled subjects were receiving either intravenous immune globulin (IVIG) or subcutaneous immune globulin (SCIG) while participating in the study. For 19 subjects, no IVIG or SCIG use was noted.

### Frequency of Poliovirus and NPEV Isolation

Thirteen patients (2%) excreted poliovirus with most patients excreting for less than 2 months following identification of infection (Table 3). The median age of poliovirus-excreting patients was 6.5 years (range 3 months–22 years). Patients with combined immunodeficiencies (three SCID and four MHC class II deficiency) were at 10-fold increased risk of excreting poliovirus (10.7%) compared to patients with predominantly antibody defects (1.0%; three CVID and three agammaglobulinemia; *p* < 0.001). The average age of patients with combined immunodeficiencies excreting poliovirus was significantly lower than in antibody deficient patients (3.3 ± 3.0 vs. 9.2 ± 7.3 years; *p* = 0.044). None of these 13 patients were coinfected with NPEV. Poliovirus excretion was not observed in any of patients with a history of paralytic disease.

**Table 3:** Data summary for all 13 PID patients with isolated poliovirus.

Patient ID	Gender	Age at study entry	Diagnosis	Site^b^	Most recent known OPV exposure	Virus	Excretion duration—no. of nucleotide changes
12-017	F	7 years	CVID	Tunisia	6 years	Sabin 1	1 month—3 nucleotide changes

12-007^a^	M	11 years	MHC II deficiency	Tunisia	1 month	Sabin 2	5 months excretion—9 nucleotide changes (0.9%)

12-025	F	7 years	MHC II deficiency	Tunisia	Unknown	Sabin 3	1 month—3 mutations

14-057	M	6 years	Agammaglobulinemia	Iran	1 week	Sabin 2	1 month—no mutations

14-108^a^	F	10 months	MHC II deficiency	Iran	6 months	Sabin 2	10 months excretion on study—12 nucleotide changes (1.2%)

14-116^a^	M	3 months	SCID	Iran	2 months	Sabin 2	6 nucleotide changes (0.6%)

14-117^a^	F	1 year	SCID	Iran	6 months	Sabin 2	10 nucleotide changes (1%)

26-012	M	22 years	CVID	Ankara, Turkey	7 years	Sabin 2	<3 months—no nucleotide changes

27-030	F	2.5 years	MHC II deficiency	Ankara, Turkey	2 years	Sabin 3	Sequence pending

20-003	M	8 months	Agammaglobulinemia	Istanbul, Turkey	6 months	Sabin 2	5 months excretion on study—3–4 nucleotide changes

11-031^a^	F	11 months	SCID	Izmir, Turkey	6 months	Sabin 3	15 nucleotide changes

19-021	M	13 years	CVID	Yekaterinburg, Russia	5 years	Sabin 3	No nucleotide changes

19-023	F	6.5 years	Agammaglobulinemia	Yekaterinburg, Russia	Not known	Sabin 2	No nucleotide changes

*OPV, oral poliovirus vaccine; CVID, common variable immune deficiency; SCID, severe combined immunodeficiency; M, male; F, female; PID, primary immunodeficiency; MHC, major histocompatibility complex*.

*^a^Patients with immunodeficiency-associated vaccine-derived polioviruses (iVDPV)*.

*^b^Countries are organized in order of World Bank income classification (lower middle and upper middle income)*.

**Figure 1:**
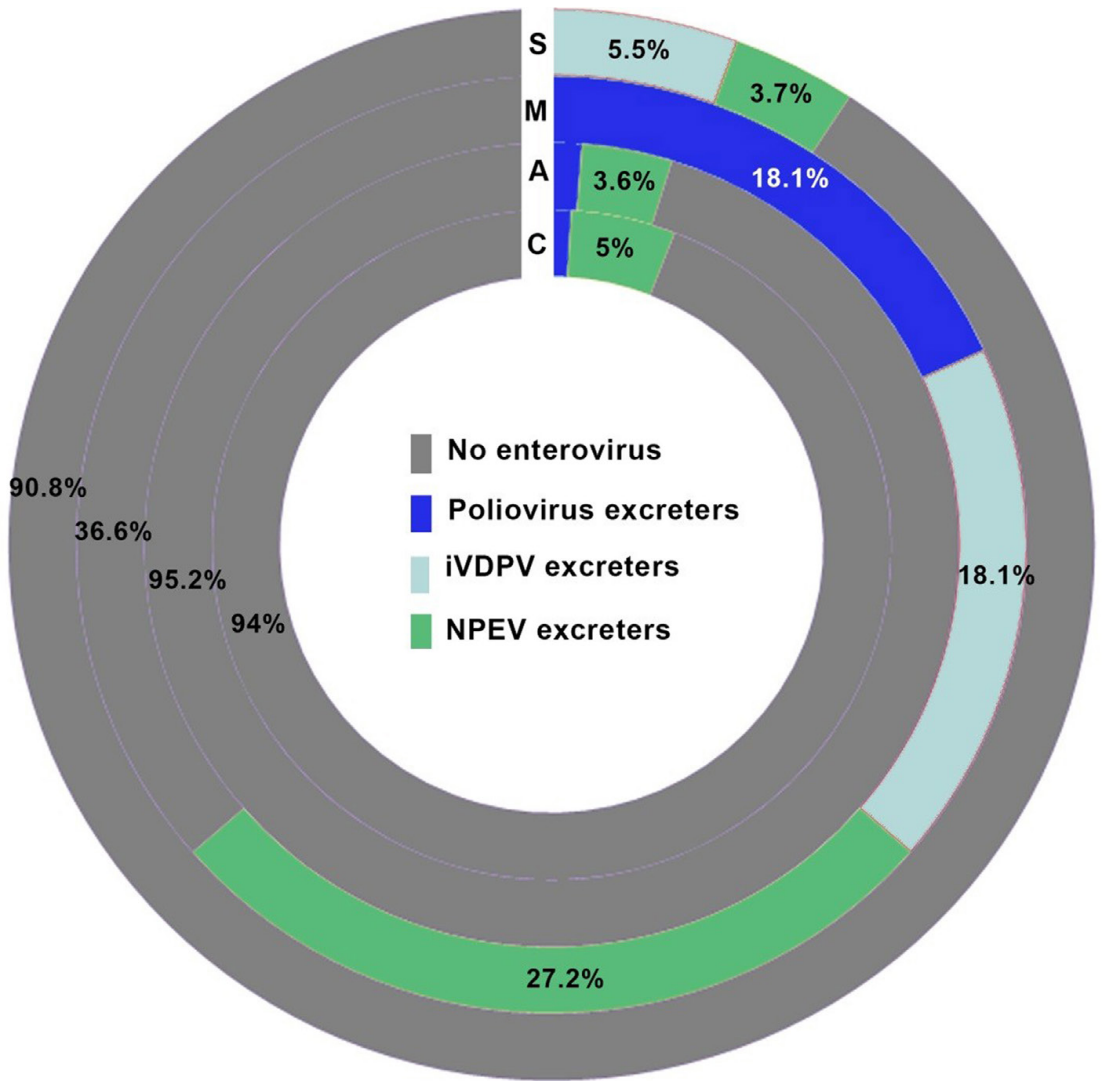
Prevalence of enterovirus excreters among different groups of primary immunodeficiency patients. C, common variable immunodeficiency; M, major histocompatibility complex II deficiency; A, agammaglobulinemia; S, severe combined immunodeficiencies.

Non-polio enteroviruses were isolated from 4.7% of evaluated patients (17 CVID, 8 agammaglobulinemia, 2 SCID, and 3 MHC class II deficiency), with a higher prevalence of NPEV infection in MHC class II deficient patients compared to patients with other forms of PIDs (*p* = 0.012; Figure 1). There was no difference in NPEV prevalence by gender (5.1% of males and 3.6% of females). Table 4 summarizes the clinical and virologic information for NPEV excreters. Echoviruses were the most commonly isolated virus (41%), followed by coxsackieviruses (24%).

**Table 4:** Data summary for all 30 PID patients with isolated non-polio enteroviruses (NPEVs).

Patient ID	Gender	Age at study entry (year)	Diagnosis	Site^b^	Class of NPEV
12-031	M	5	MHC II deficiency	Tunisia	NA
12-037	M	0.4	MHC II deficiency	Tunisia	NA
12-006^a^	M	5	CVID	Tunisia	Negative/E2

15-014	M	10	XLA	Argentina	E25
23-003	M	7	Agammaglobulinemia	China	NA
23-004	M	14	Agammaglobulinemia	China	NA
24-020	M	22	CVID	Columbia	NA
24-023	M	4	Agammaglobulinemia	Columbia	NA
14-079	F	28	CVID	Iran	EV11
14-020	M	7	Agammaglobulinemia	Iran	EV20
14-019	M	41	CVID	Iran	EV6
14-121	M	17	CVID	Iran	NA
25-020	M	8	CVID	Mexico	NA
25-019^a^	M	8	Agammaglobulinemia	Mexico	NA
27-021	F	2	MHC II deficiency	Ankara, Turkey	CA5/no second sample
20-025	M	1	Agammaglobulinemia	Istanbul, Turkey	EV9
20-007	F	18	CVID	Istanbul, Turkey	CA4
20-006	M	20	CVID	Istanbul, Turkey	EV 33
11-018	M	5	SCID	Izmir, Turkey	CA10
11-050	F	6	CVID	Izmir, Turkey	CA2
11-048	M	0.5	CVID	Izmir, Turkey	CB
11-042	M	11	CVID	Izmir, Turkey	CB4
11-016	M	17	CVID	Izmir, Turkey	E6
11-075	F	0.7	CVID	Izmir, Turkey	NA
11-068	M	7	CVID	Izmir, Turkey	E7

29-012	F	3	Agammaglobulinemia	Israel	CB5
29-006	F	12	SCID	Israel	EV01
29-016	M	5	CVID	Israel	EV11
29-024	M	20	Agammaglobulinemia	Israel	EV13
29-008	M	9	Agammaglobulinemia	Israel	NA

*OPV, Oral poliovirus vaccine; CVID, common variable immune deficiency; SCID, severe combined immunodeficiency; M, male; F, female; CA, coxsackie A virus; CB, coxsackie B virus; EV, enterovirus; E, echovirus; PID, primary immunodeficiency; MHC; major histocompatibility complex*.

*^a^Samples were collected prior to immunoglobulin replacement therapy*.

*^b^Countries are organized in order of World Bank income classification (lower middle, upper middle, and upper income)*.

The mean age of poliovirus excreting patients was significantly lower than NPEV excreters (5.9 ± 4.3 vs. 10.4 ± 9.2 years, *p* = 0.005). Samples from nine patients (1.4% of 635 studied patients) were collected at the time of PID diagnosis and prior to administration of immunoglobulin replacement therapy. Of note, two of these nine cases (22.2%) were NPEV positive, both were diagnosed with antibody deficiency and were from the same center (12-006 and 12-019; *p* = 0.06). No poliovirus was isolated from these nine patients.

### Predominance of iVDPV among Combined Immunodeficiency

Five (0.8% of all patients) of 13 poliovirus-excreting patients were considered iVDPV excreters based on the number of VP1 nucleotide changes (median of 10 nucleotide changes; range, 6–15). Of note, all iVDPV excreters had combined immunodeficiency (three SCID and two MHC class II deficiency), and four of these five patients excreted Sabin 2 strains (Table 3). The median number of mutations did not differ between SCID (1%; range 0.6–1.5%) and MHC II deficiency (1.05%; range, 0.9–1.2%) patients. None of the iVDPV excreters presented with acute flaccid paralysis, but chronic diarrhea was a common clinical manifestation. OPV vaccination had occurred in all of iVDPV excreters, and the median time from the first OPV dose to identification of iVDPV was 10 months (range 3 months–11 years). The sequence divergence suggests that secondary exposure may have been the source of infection in some cases. Further vaccination was avoided in all patients due to the diagnosis of PID.

### Clinical Presentation and Genetic Studies of iVDPV Excreters

One MHC class II deficiency patient (12-007) was followed for several years prior to enrollment in the current study showing intermittent VDPV excretion episodes and reinfection with various VDPV strains and with several NPEV from a sibling and from the community (17). During the current study, he was infected with Sabin 2 strain (Table 3). The second MHC class II patient (14-108) was a 10-month-old girl with disseminated granulomatous disease (BCGosis) after BCG vaccination at age 4 months who excreted poliovirus for 5 months. The mutation in *CIITA* gene (homozygous c.3242-3244delACA, p.Asn1082del) was confirmed in this patient using targeted PID genes panel sequencing.

The only Sabin serotype-3 iVDPV excreter was a girl with SCID (11-031; *RAG1* c.1682G>A, p.R561H) enrolled at age 11 months. This poliovirus had the highest number of nucleotide changes (15 nucleotides) observed in the study. Virus elimination was evident 3 months after hematopoietic stem cell transplantation. Two other SCID patients infected by serotype-2 iVDPV had the molecular diagnosis confirmed with a mutation in *ADA* (14-116; homozygous c.415G>T, p.Glu139X) and a mutation in *RAG1* (14-117; homozygous c.1180C>T, p.Arg394Trp) genes, respectively.

## Discussion

Primary immunodeficiency patients having defects in humoral or cellular immunity are at risk of chronic/prolonged infection with enteroviruses, including polioviruses. These patients can excrete iVDPV after receiving OPV or after being exposed to a household or community contact excreting poliovirus. Patients who are excreting polioviruses are at risk of developing paralytic poliomyelitis. Such patients must be identified, and their infection resolved, to protect the patients and achieve global eradication of poliovirus. The present study brings together the JMF network, TFGH, WHO, and CDC to estimate the prevalence of poliovirus excretion in selected PID patients who have been exposed to OPV. Two of the participant countries are high income (Israel and Japan); the remainder are lower (two countries) or upper (seven countries) middle income countries according to the World Bank classification. Three poliovirus excreters were from a lower middle income country (Tunisia), and the remainder were from upper middle income countries. NPEVs were isolated from patients from countries from all three economic strata.

Although few cross-sectional studies have been conducted to identify iVDPV among PID patients without paralysis (12, 17–21), this study identified the largest number of PID patients both with antibody and combined immune deficiencies. This study also constituted a large number of countries with worldwide distribution from four continents (Asia, Africa, Europe, and South America). Approximately 2% of the studied PID patients excreted poliovirus. PID survival rates are improving in lower and middle income countries (22). Furthermore, iVDPV excreters are being identified at a markedly increased rate due to improved surveillance that includes polio symptom-free excreters (3). This reinforces the need for enhanced surveillance of PID patients until polio eradication is certified and the use of OPV is stopped. The findings of this study could help estimate the prevalence of iVDPV excreters, which is needed to assess the global risk to eradication posed by these excreters. Undiagnosed PID patients and cases with delayed diagnosis should be considered as potential contributors to underestimation.

In 5% of the studied patients, NPEV was isolated, and none of them were concomitantly excreting poliovirus. Although this prevalence was lower than in a previously reported single-center study (20%) (12), it demonstrates the susceptibility of patients with humoral and cellular immunodeficiency to prolonged excretion of all enteroviruses. Higher prevalence of NPEV compared to OPV strains might be due to the multiplicity of circulating NPEV serotypes. Moreover, immunoglobulin preparations used for treatment of PID patients do not contain high titers of immunoglobulin against all NPEVs. Of note, immunoglobulin replacement might contribute to the relatively short-term excretion of OPV strains observed in some PID patients; however, we could not test this phenomenon as all poliovirus excreters in this study were receiving IVIG or SCIG treatment.

Poliovirus 2 was the most prevalent serotype detected in iVDPV excreting PID patients (80%), followed by serotype 3 (20%); consistent with the findings in PID patients with paralysis (4, 8). Concurrent infection with more than one iVDPV serotype has been documented in other studies; but we did not observe simultaneous shedding of more than one iVDPV serotype in any patient. Possible explanations for the predominance of poliovirus 2 include the high frequency of recombination that can occur with poliovirus 1 and 3 in OPV vaccinated individuals, resulting in a virus with a higher replication efficiency (23–26). Sabin poliovirus 2 also replicates longer and is transmitted more readily due to greater replication fitness or the ability to out-compete the other two serotypes for the binding to the CD155 receptor in the host cells (27, 28). Although these characteristics are potential factors for an increased risk of iVDPV2 in PID, they contributed to the eradication of WPV type 2 (29) and poliovirus 2 has since been removed from the trivalent OPV vaccine (30).

All SCID poliovirus excreters and 50% of MHC class II deficient poliovirus excreters became iVDPV excreters. While the number of iVDPV excreters was small, this result suggests that cellular immunity may contribute effectively to clearance of enterovirus infections including cessation of poliovirus excretion. Both humoral (antigen-specific plasma cells and memory B cells) and cellular (memory T cells) immunities should be developed following an effective immunization especially for complex vaccines against viruses. In addition to the capability of OPV-infected dendritic cells to engage specific CD8 T cells, the presence of OPV-specific CD4 T cells in vaccinated individuals is crucial. CD4 T cells are involved in serotype-specific antibody production (B cell priming by follicular helper T cells) and interferon gamma production to lyse infected target cells (virus clearance by cytolytic effector CD4 cells as well as cytolytic effector CD8 T cells). Antigen presentation in both functions of CD4 T cells is through MHC class II (31). Therefore, patients with combined immunodeficiencies who lack both humoral and cellular immunities may be more likely to be unable to stop virus excretion. However, the presence of six patients with antibody deficiency with long-term poliovirus shedding (approximately 9 years) highlights the important role of viral specific-antibodies in complete clearance of the virus (32). Considering the several reports of antibody deficient patients with paralysis caused by iVDPV, the identification and treatment of poliovirus symptom-free virus excreting patients is necessary (3–5).

Wild poliovirus or iVDPV infections were not observed in the patients with a history of the paralytic disease, and none of their neurologic symptoms were linked to poliovirus infection. Moreover, paralytic disease in the context of PID has several potential etiologies including neurologic autoimmune disorders including multiple sclerosis, myalgic encephalomyelitis, myasthenia gravis, Guillain–Barré syndrome, chronic inflammatory demyelinating polyneuropathy, and vasculitic neuropathies. Moreover, vitamin deficiency (B12) and paraneoplastic neuropathy should be considered as differential diagnosis for paralysis in PID patients (33).

This study provides an estimate of the global iVDPV prevalence among PID patients without paralytic disease and supports expanded screening for iVDPV excretion in these patients. Although most previous studies focused on the risk of long-term iVDPV infection in antibody deficient patients, the predominance of risk in patients with combined immunodeficiencies included in the current study highlights the importance of considering this group of PID patients in any surveillance program. Reinfection with poliovirus and NPEV excretion in PID patients described elsewhere demonstrates the need for prolonged follow-up (17).

The Global Polio Eradication Initiative plans to cease use of OPV worldwide once WPV has been certified as eradicated, which will end the generation of new iVDPVs. However, there is currently no means for addressing the threat posed by existing immunodeficient persons infected with iVDPVs, either to the infected individual’s risk of paralytic disease, or to the community of a continuing source of poliovirus transmission. Antivirals represent a potential means to manage the treatment of iVDPV excreters and the risk they present to the eradication effort (32, 34). Two safe virus-specific antivirals acting by differing mechanisms are now being developed and may be used as a combination (e.g., pocapavir and V-7404). This strategy may resolve the individual’s infection, stop iVDPV excretion, and serve to eliminate the risk of poliovirus transmission in the community. Currently, pocapavir is being considered for use in poliovirus excreting PID patients on a compassionate use basis.

The limitations of the existing study include that no low-income level country participated, which may bias the generalization of these findings. Moreover, other forms of less profound combined immunodeficiency should be evaluated in future studies since this study recruited only patients with MHC class II deficiency.

The potential risk posed by iVDPV excreters to the polio eradication effort indicates the immediate need to develop and implement a global iVDPV surveillance strategy. Utilizing this approach, individuals at risk of prolonged poliovirus excretion can be identified and antiviral treatment can be initiated.

## The JMF Centers Network Investigators and Study Collaborators

The following JMF centers network investigators and study collaborators contributed to the conduct of this study.

**Ahmet Ozen**, Division of Pediatric Allergy and Immunology, Marmara Medical Faculty, Istanbul, Turkey; **Andrea Berlin**, Center for Vaccine Equity, The Task Force for Global Health, Decatur, GA, United States; **Anissa Chouikha**, Department of Virology, Institut Pasteur de Tunis and University Tunis El-Manar, Tunis, Tunisia; **Armando Partida-Gaytán**, Immunodeficiency Research Unit, Instituto Nacional de Pediatría, Ciudad de México, Mexico; **Ayca Kiykim**, Division of Pediatric Allergy and Immunology, Marmara Medical Faculty, Istanbul, Turkey; **Charu Prakash**, Division of Microbiology, National Centre for Disease Control, New Delhi, India; **Deepti Suri**, Allergy Immunology Unit, Advanced Pediatrics Centre, PGIMER, Chandigarh, India; **Deniz Cagdas Ayvaz**, Division of Immunology, Department of Pediatrics, Hacettepe University Faculty of Medicine, Ankara, Turkey; **Dioselina Peláez**, Grupo de Virología, Instituto Nacional de Salud, Bogotá, Colombia; **Edson Elias da Silva**, Enterovirus Laboratory, Oswaldo Cruz Institute, Oswaldo Cruz Foundation, Rio de Janeiro, Brazil; **Ekaterina Deordieva**, Department of Clinical Immunology, Dmitry Rogachev Federal Research and Clinical Center of Pediatric Hematology, Oncology and Immunology, Moscow, Russia; **Elda Edith Pérez-Sánchez**, Poliovirus Lab – Instituto de Diagnóstico y Referencia Epidemiológicos, Secretaría de Salud, Ciudad de México, Mexico; **Ezgi Ulusoy**, Faculty of Medicine, Department of Pediatric Immunology, Ege University, Izmir, Turkey; **Figen Dogu**, Department of Pediatric Immunology and Allergy, Ankara University School of Medicine, Ankara, Turkey; **Gisela Seminario**, Hospital de Niños Dr. Ricardo Gutierrez, Buenos Aires, Argentina; **Hacer Cuzcanci**, Division of Immunology, Department of Pediatrics, Hacettepe University Faculty of Medicine, Ankara, Turkey; **Hinda Triki**, Department of Virology, Institut Pasteur de Tunis and University Tunis El-Manar, Tunis, Tunisia; **Hiroyuki Shimizu**, National Institute of Infectious Diseases, Tokyo, Japan; **Ilhan Tezcan**, Division of Immunology, Department of Pediatrics, Hacettepe University Faculty of Medicine, Ankara, Turkey; **Imen Ben-Mustapha**, Department of Immunology, Institut Pasteur de Tunis and University Tunis El-Manar, Tunis, Tunisia; **Jinqiao Sun**, Department of Clinical Immunology, Children’s Hospital of Fudan University, Shanghai, China; **Juliana T. Lessa Mazzucchelli**, Department of Pediatrics, Federal University of São Paulo, São Paulo, Brazil; **Julio César Orrego**, Grupo de Inmunodeficiencias Primarias, Departamento de Microbiología y Parasitología, Facultad de Medicina, Universidad de Antioquia UdeA, Medellín, Colombia; **Małgorzata Pac**, Department of Clinical Immunology, The Children’s Memorial Health Institute, Warsaw, Poland; **Mikhail Bolkov**, Institute of Immunology and Physiology, Ural Branch of the Russian Academy of Sciences, Yekaterinburg, Russia; **Mónica Giraldo**, Grupo de Inmunodeficiencias Primarias, Departamento de Microbiología y Parasitología, Facultad de Medicina, Universidad de Antioquia UdeA, Medellín, Colombia; **Nabil Belhaj-Hmida**, Department of Immunology, Institut Pasteur de Tunis and University Tunis El-Manar, Tunis, Tunisia; **Najla Mekki**, Department of Immunology, Institut Pasteur de Tunis and University Tunis El-Manar, Tunis, Tunisia; **Natalia Kuzmenko**, Department of Clinical Immunology, Dmitry Rogachev Federal Research and Clinical Center of Pediatric Hematology, Oncology and Immunology, Moscow, Russia; **Neslihan E. Karaca**, Faculty of Medicine, Department of Pediatric Immunology, Ege University, Izmir, Turkey; **Nima Rezaei**, Research Center for Immunodeficiencies, Pediatrics Center of Excellence, Children’s Medical Center, Tehran University of Medical Science, Tehran, Iran; **Ousmane Madiagne Diop**, Global Laboratory Network Coordinator, World Health Organization, Geneva, Switzerland; **Safa Baris**, Division of Pediatric Allergy and Immunology, Marmara Medical Faculty, Istanbul, Turkey; **Sau Man Chan**, Department of Paediatrics and Adolescent Medicine, The University of Hong Kong, Hong Kong Special Administrative Region, Hong Kong, China; **Shohreh Shahmahmoodi**, Virology Department, School of Public Health, Tehran University of Medical Sciences, Tehran, Iran; **Sule Haskologlu**, Department of Pediatric Immunology and Allergy, Ankara University School of Medicine, Ankara, Turkey; **Wenjing Ying**, Department of Clinical Immunology, Children’s Hospital of Fudan University, Shanghai, China; **Ying Wang**, Department of Clinical Immunology, Children’s Hospital of Fudan University, Shanghai, China.

## Ethics Statement

This study was approved by Western Institutional Review Board.

## Author Contributions

AA, HA, and MRB performed clinical investigation, analyzed data, and wrote the manuscript. MM, MP, SW, RS, SK, JQ, FM, and VM were responsible for conception and design of the study, interpreted data, and supervised the study. NK, XW, OS, TL, AI, EB, IT, BC-C, JF, RS, EK-A, SS, LB, FE-R, AS, Y-LL, and SN recruited patients and performed clinical investigation. All the authors read and approved the final manuscript. Task Force for Global Health prepared the protocol, developed the study, and coordinated study implementation and reporting. US Centers for Disease Control and Prevention sequenced isolated enterovirus and managed database of patients. World Health Organization Global Polio Laboratory Network developed standard operating procedures, study forms, and training material and analyzed stool samples for presence of enterovirus. Jeffrey Modell Foundation selected clinical sites and facilitated clinical investigator enrollment and communication with patients.

## Conflict of Interest Statement

The authors declare that the research was conducted in the absence of any commercial or financial relationships that could be construed as a potential conflict of interest.

## Footnotes

^1^http://esid.org/Working-Parties/Registry/Diagnosis-criteria.

^2^http://www.who.int/immunization/monitoring_surveillance/burden/vpd/surveillance_type/active/poliomyelitis_standards/en/.
