# Supplementary material for: Patients with Primary Immunodeficiencies Are a Reservoir of Poliovirus and a Risk to Polio Eradication
Source: Front Immunol. 2017 Jun 13;8:685. doi: 10.3389/fimmu.2017.00685 (PMC5468416; doi:10.3389/fimmu.2017.00685)
Supplement: Supplementary file 1 [file data_sheet_1.docx]

**SUPPLEMENTAL DATA**

**Patients with Primary Immunodeficiencies are a Reservoir of Poliovirus and a Risk to Polio Eradication**

Aghamohammadi et al.

**Table S1-** Criteria used for clinical diagnosis of the studied PID patients

| **Diagnosis** | **Clinical criteria** |
| --- | --- |
| Combined  immunodeficiency  (CID) | At least one of:  - At least one severe infection (requiring hospitalization)  - One manifestation of immune dysregulation (autoimmunity, severe eczema, lymphoproliferation, granuloma)  - Malignancy  - Affected family member  AND 2 of 4 T cell criteria fulfilled:  - Reduced CD3 or CD4 or CD8 T cells  - Reduced naive CD4 and/or CD8 T cells  - Elevated g/d T cells  - Reduced proliferation to mitogen or T cell receptor stimulation  AND HIV excluded |
| Severe non-syndromic combined immunodeficiency (SCID) | CID  At least one of the following:  - Invasive bacterial, viral or fungal/opportunistic infection  - Persistent diarrhea and failure to thrive  - Affected family member  AND manifestation in the first year of life  AND Absence or very low number of T cells (CD3 T cells < 300/ul) |
| Common variable immunodeficiency (CVID) | At least one of the following:  - increased susceptibility to infection  - autoimmune manifestations  - granulomatous disease  - unexplained polyclonal lymphoproliferation  - affected family member with antibody deficiency  AND marked decrease of IgG and marked decrease of IgA with or without low IgM  levels (measured at least twice; <2SD of the normal levels for their age);  AND at least one of the following:  - poor antibody response to vaccines (and/or absent isohaemagglutinins);  i.e. absence of protective levels despite vaccination where defined  - low switched memory B cells (<70% of age-related normal value)  AND secondary causes of hypogammaglobulinaemia have been excluded  AND diagnosis is established after the 4th year of life (but symptoms may be present  before)  AND no evidence of profound T-cell deficiency |
| Agammaglobulinemia | Fewer than 2% circulating B cells (CD19 and CD20), preferably in two separate  determinations and a normal number of T cells (CD3, CD4 and CD8)  AND serum IgG levels below:  -200 mg/dl in infants aged < 12 months  -500 mg/dl in children aged > 12 months  OR normal IgG levels with IgA and IgM below 2SD  AND onset of recurrent infections before 5 years of age  AND secondary causes of hypogammaglobulinaemia have been excluded  OR positive maternal family history of agammaglobulinaemia |

**Table S2-** Possible options for collection of stool samples depending on the proximity of the patient to the JMF study site, the practicality of patient travel to the study site and the availability of adequate cold storage of the stool.

| **ID** | **Proximity of the patient to JMF site** | **Practicality of patient travel** | **Availability of adequate cold storage** | **Possible options for first samples** | **Possible options for second samples** |
| --- | --- | --- | --- | --- | --- |
| 1 | Near | Easy | No | Patient provided stool sample during enrolment visit. | One additional sample once over the subsequent 3 day period. |
| 2 | Near | Easy | Yes | Patient provided a stool sample during the enrolment visit and returned home with collection kit and adequate cold storage. | Second stool sample was collected over the subsequent 3 days and was returned to the study site as directed. |
| 3 | Far | Difficult | No | Patient provided a stool sample during the enrolment visit and was housed overnight near the study site. | Provides a second sample at the study site the next day. |

## English Version of Consent form

**Consent to be in a Study to Determine the Prevalence of Poliovirus Excretion in Immune Deficient Individuals**

Your treating physician and the Task Force for Global Health invite you to be in a research study.

**TITLE:** Study to Determine the Prevalence of Poliovirus Excrection in Immune Deficient Individuals

**PROTOCOL NO.:** CVE-001

WIRB® Protocol #20130957

**SPONSOR:** The Task Force for Global Health

**INVESTIGATOR:** Name

Address

City, State Zip

Country

**SITE(S):** Name

Address

City, State Zip

Country

**STUDY-RELATED**

**PHONE NUMBER(S):** Name

Phone Number(s)

In this consent form, “you” always refers to the subject. If you are a parent or guardian, please remember that “you” refers to the study subject.

**Background**

Poliovirus infection (poliomyelitis, or polio) may cause paralysis of the legs, arms or the muscles for breathing and can result in death. The oral polio vaccine (OPV) given to children contains safe polioviruses, although very rarely OPV can cause polio disease in healthy children. On rare occasions in a community with low vaccination levels, this vaccine virus can spread over time and change to vaccine-derived poliovirus (VDPV) and cause polio disease. Individuals with an inability to develop body defenses against infections (primary immunodeficiencies [PI]) may have a problem fighting infections due to viruses. If individuals with PI had received OPV or if they were exposed to a person who had received OPV, they can excrete VDPV over a prolonged period and are at increased risk for developing polio many years afterward. Further, these patients must be identified, and their infection resolved if possible, in order to ensure the global eradication of poliovirus.

The Poliovirus Antivirals Initiative at the Task Force for Global Health (TFGH) is a collaboration of governmental and non-governmental organizations and industry sponsors dedicated to the development of poliovirus antiviral drugs with the potential to stop virus excretion by individuals with PI. CDC is a major partner of the Global Polio Eradication Initiative, and has provided fundamental scientific support to the initiative, including the development of potential polio antiviral products. The Jeffrey Modell Foundation (JMF) was established to identify and advocate for PI patients. JMF has built a network of over 500 PI treating physicians in 191 cities in 64 countries. The World Health Organization (WHO) has a global network of 146 qualified laboratories to assay for poliovirus. WHO also has conducted VDPV surveillance primarily focused on children with acute flaccid paralysis. The present research study will bring together the JMF network with the CDC, TFGH and WHO to determine the frequency of PI patients who may be excreting poliovirus. PI patients who are excreting poliovirus may be invited to participate in subsequent research on poliovirus antiviral(s) currently in development. These antivirals may or may not have activity against shedding of non-polio enteroviruses.

**Why were you invited to participate in the study?**

We are talking to you about this research study because you (or your child) have (has) a primary immunodeficiency that puts you at risk of continued excretion of polio vaccine virus. If you are excreting vaccine virus then you are at a risk of developing paralytic disease. You may then be invited to participate in a subsequent research study of an antiviral drug, which may help stop the virus from being excreted. If this happens, at a future date you will be fully informed in writing of the potential risks and benefits of this drug before providing consent for treatment by the collaborators in that research.

**Methods and procedures**

You will be asked to discuss this study with your treating physician. If you decide to participate in this study, you will be given a specimen collection kit with instructions for obtaining stool specimens and will review the procedures with your physician or a representative of the physician’s office. You will be requested to collect about 8 grams (about the size of one adult thumb) of stool on two separate days over a four-day period. Your physician’s office will arrange transport of your samples to the laboratory for testing. If you are required to travel to the clinical site, you will be reimbursed for lodging and meals at a nearby facility. After collection of the stool samples, you or your physician’s office will keep it at refrigerator temperature (2-8 degree Celsius). You will be asked to permit storage of stool specimens for no more than 5 years for potential future use in studies. If you are found to be excreting virus, your treating physician and the WHO laboratory may continue to monitor the excretion until it ceases, which occasionally happens spontaneously.

**Payment**

You will be paid $75 for your participation to cover incidental expenses and we will cover the cost of travel, lodging, and food.

**Risks and benefits**

There is no significant risk to stool collection. The benefit of participation is that you will learn if you are excreting poliovirus or non-polio enterovirus and can take additional measures to prevent its spread.

**Alternatives**

Your alternative is to not be in this study.

**Privacy, anonymity and confidentiality**

Only a few persons working in the study at the physician’s office will have access to identifying information about you or your child such as your name, address and contact information. They will need this information to contact you to give you the results of the analysis. Some confidential information will be collected about you and your immune deficiency for analysis that will not be linked with your identifying information. Other people helping with the study will only get general information that is not linked to your name or other personal information. We will try very hard to limit access to records that could identify any person in the study. Stool samples will include only coded numbers. The persons who test the samples will not have information that could identify you or your child. If the results of this study are published you or your child will not be identified by name in any published documents.

The information may also be given to the governmental agencies in this and other countries. Research records which identify you and the consent form signed by you may also be looked at and/or copied for research or regulatory purposes by:

- Department of Health and Human Services (DHHS) agencies,
- the institution where the research is being done, and
- Western Institutional Review board® (WIRB®).

**Right not to participate and withdraw**

Your consent for you or your child to take part in this study is voluntary. You do not have to take part in this study if you do not wish to do so. You may decline to take part now or stop taking part at any time. If you refuse to take part or withdraw from the study later, there won’t be any penalty nor will you or your child lose any access to medical services or other benefit.

Your or your child’s participation in this study may be stopped at any time by the study doctor or the sponsor without your consent for any reason.

**Contact information for more information**

You can ask any questions about this study or the consent form at any time. If you have questions, concerns, or complaints about the study you may speak to:

Name of local investigator: _ ________Phone: _

Name of principal investigator: _ ________Phone: _

If you have questions about you or your child’s rights as a participant in this research study you may contact:

Western Institutional Review Board® (WIRB®):

3535 Seventh Avenue, SW

Olympia, Washington 98502

Telephone: 1-800-562-4789 or 360-252-2500

E-mail: [Help@wirb.com](mailto:Help@wirb.com)

WIRB is a group of people who perform independent review of research.

WIRB will not be able to answer some study-specific questions, such as questions about appointment times. However, you may contact WIRB if the research staff cannot be reached or if you wish to talk to someone other than the research staff.

If you think that your child may have been harmed during this study, you may also contact a study investigator at the Task Force for Global Health:

Name: __________ ____________ Phone: ___________________

If you agree to enroll you or your child in the study, please indicate that by putting your signature or your left thumb impression at the specified space below.

Thank you for your cooperation

I agree for myself ( ) or my child ( ) to participate.

Consent for additional laboratory testing on stored stool specimens: Check the box and initial your choice

I agree to allow myself or my child’s stored stool specimens to be used for additional laboratory testing.

I do not want my or my child’s stored stool specimens to be used for additional laboratory testing

***Consent and Assent Instructions:***

*Consent: Subjects 18 years and older must sign on the subject line below*

*For subjects under 18, consent is provided by the parent or guardian*

*Assent: Is not required for subjects 6 years and younger*

*Verbal assent is required for subjects ages 7 through 12 years using the Assent Form*

*Written assent is required for subjects ages 13 through 17 years using the Assent Form*

______________________________________

Signature or left thumb impression Date

Name of the subject: ___________________________________

Name of parent / guardian: ___________________________________

(If not the subject) Relationship: ___________________________________

_______________________________________

Signature of the witness Date

_______________________________________

Signature of the Clinical Investigator Date

**ASSENT SECTION:**

Statement of person conducting assent discussion:

- I have explained all aspects of the research to the subject to the best of his or her ability to understand.
- I have answered all the questions of the subject relating to this research.
- The subject agrees to be in the research.
- I believe the subject’s decision to enroll is voluntary.
- The study doctor and study staff agree to respect the subject’s physical or emotional dissent at any time during this research when that dissent pertains to anything being done solely for the purpose of this research

_________________________________ _____________________

Signature of Person Conducting Date

Assent Discussion

Statement of Parent or Guardian:

My child appears to understand the research to the best of his or her ability and has agreed to participate.

___________________________ ______________________________

Signature of Parent or Guardian Date

Physician / Investigator / Designee statement

I hereby certify that I have discussed the research project with the subject or parent / guardian of the research participant. I have explained all the information contained in the informed consent document, including any risks that may be reasonably expected to occur. I certify that the research participant was encouraged to ask questions and that all questions were answered.______________________________ ________________

Physician / Investigator / Designee Date

**English Version of Assent form**

**For children 7-17 years of age**

**Assent to be in a Study of Poliovirus Excretion in Immune Deficient Individuals**

**TITLE:** Study to Determine the Prevalence of Poliovirus Excretion in Immune Deficient Individuals

**PROTOCOL NO.:** CVE-001

WIRB® Protocol #20130957

**SPONSOR:** The Task Force for Global Health

**STUDY- RELATED**

**PHONE NUMBER(S):**

Your treating physician and the Task Force for Global Health invite you to be in a research study.

***What is the purpose of this study?***

People who cannot develop body defenses against infections (primary immunodeficiencies [PI]) may have a problem fighting infections due to viruses. If PI patients had received oral poliovirus vaccine (OPV) or if they were exposed to a person who had received OPV, they can occasionally shed poliovirus in their stools over a prolonged period. If this is the case, they have a risk of developing paralytic polio.

***How much time will it take for me?***

You are asked to spend about a half-hour to have your parents or guardians and you understand what the study is about, and then spend the time needed for two stool specimens to be collected over a four-day period.

***What do you want me to do if I decide to be in this study?***

If you decide to be in this study, you will be given a specimen collection kit with instructions for obtaining stool specimens. You and your parents or guardians will review the procedures with someone from the physician’s office. You will be requested to collect stool samples on two separate days over a four-day period. These samples will be sent to the laboratory for testing. If you traveled some distance to the clinical site, you and your parents or guardians will be given lodging and meals nearby.

***Are there any risks to me if I decide to be in this study?***

There is no significant risk to stool collection.

***Are there any benefits from being in this study?***

You will learn if you are shedding poliovirus in stools. If you are found to be shedding, you have some risk of developing polio. Knowing if you shed poliovirus may allow you to get treatment in the future.

***Who should I call if I have questions about this?***

If you have questions about this study you can talk to your treating physician.

***Do I have to be in this study?***

You do not have to take part in this study if you do not wish. You may decline to take part now or stop taking part at any time. If you refuse to take part in the study you will not lose any of your medical care.

***Signature***

I was told about the study. I asked questions. I had my questions answered. I want to be in the study.

______________________________ ________________

Signature of child (13-17) Date
